# The Operative Treatment of Inguinal Hernia in Hospital

**Published:** 1917-03-31

**Authors:** Arthur A. Straton

**Affiliations:** Temp. Capt. R.A.M.C.


### THE OPERATIVE TREATMENT OF INGUINAL HERNIA IN HOSPITAL.

By ARTHUR A. STRATON, M.D. Lond., F.R.C.S. Edin. (Temp. Capt. R.A.M.C.)

From a variety of causes during the past decade the number of cases of hernia presenting themselves for operation at the various hospitals throughout the country has become yearly more and more formidable, and as a consequence the supply of beds for these patients has become one of the problems of hospital administration.

A glance at the "waiting-list" of any large hospital, metropolitan or provincial, will speedily convince the inquirer as to the need of the greatest economy of time in dealing with these cases; a single unnecessary day spent in hospital when applied to each bed in the surgical wards, if only occurring but twice in the year, means—for a service of fifty surgical beds—a waste of no less than fourteen weeks of possible occupation, or time enough, on a generous estimate, for seven cases of hernia to receive their radical cure. Applied to a service of one hundred beds, and allowed to occur five or six times in the course of a year, the amount of wastage becomes stupendous.

The management of cases of hernia in hospital is a matter that rests largely with the house surgeon in charge of the beds, since in these days of great operative activity it is usually the resident who performs the greatest number of operations for radical cure during the year, and for this reason a great deal depends upon the methods that he may employ.

### The Management of Beds.

The admission of cases is usually his business entirely, so that he should never fail to arrange for his beds to be refilled as soon as they become vacant; and in order to secure this result he should be able to keep a plan of his beds in his "mind's eye," together with the probable date of each vacancy. Further, he should try to ensure his patients' arrival on the date arranged, by writing them at least three days ahead of this day, so that they may have sufficient time to warn their employers and to arrange for the upkeep of their households during their absence.

In the management of beds it is the wisest policy to keep a certain definite proportion free for cases of emergency; and by taking the weekly average of emergencies admitted during the previous year one is enabled to arrive at a fair allowance necessary for such cases. Although this diminishes the number of beds available for cases upon the waiting-list, yet such an allowance is in reality a necessity and should be insisted upon in every service of every hospital, as being the only means of ensuring smooth working among the residents, of abolishing the continual nuisance of borrowing beds, and further of avoiding the overcrowding of the wards, and the overworking of their staffs that must inevitably ensue where such a rule is not enforced.

With regard to hernia-patients in particular there are certain points to be borne in mind as being desirable in the treatment of each case; these are as follows:—

(i) A minimum pre-operative stay in hospital.

(ii) The operation should be thorough, but as rapid as may be consistent with good workmanship and efficient results.

(iii) A minimum post-operative stay in hospital as far as may be consistent with the avoidance of recurrences.

Only by observing the above postulates may a true economy of beds and of time be obtained.

### Pre-operative Treatment.

The actual admission of the patient should be arranged for the day previous to the operation, preferably after the midday meal, in the early afternoon. He should have been directed to shave his pubic region thoroughly the same morning; where this has been omitted he should be shaved by the barber immediately upon admission. He should have a thorough hot bath before retiring, and should receive an effective dose of a good purgative, strong enough to ensure action without fail, as soon as possible after arrival in the ward, preferably from eighteen to twenty-four hours before operation.

The complicated preparations of ten years ago, comprising a shave by a tired-out house-surgeon or dresser, usually at night, followed by applications of ether soap, turpentine and ether, and culminating in the bandaging over the operation area of a chill, dank compress soaked in some antiseptic, are nowadays generally admitted to be more of a danger than a benefit to the patient, in that they render the skin sodden and open to infection. Even the application of a sterile dry compress to prevent external contamination is really unnecessary, and is usually rendered futile by the patient removing it whilst at stool.

The application, whilst the patient is upon the operating table, of iodine, preferably followed by a solution of perchloride of mercury in 70 per cent. spirit, provides an ample security for aseptic healing of the wound in cases of hernia, which are admittedly among the most frequent to become septic; and this is made manifest by the fact that emergency cases of strangulation taken directly to the theatre without previous preparation, and there shaved and treated by the above method, show no appreciably greater proportion of sepsis than those cases prepared in the most careful way the day previous to operation. It only requires a comparison of healing records in such cases to demonstrate the truth of this assertion, and such may be made at any large hospital adopting the iodine preparation.

### An "Iniquitous Practice."

Another procedure of the past generation that has also been proved unnecessary in the ordinary case of radical cure is the iniquitous practice of administering one and sometimes two enemata to every operation case, usually arousing the patient in the early hours of the morning to perform these exercises upon him. A good purgative eighteen to twenty-four hours before operation is ample provision in this respect, and the enema is a method of torture that should be reserved for those whose bowels have proved refractory to ordinary measures, since the shock and pain produced by the usual two-pint injection of soap and water are quite sufficient to reduce to its lowest level the resistance of the patient, just at the time when he has the greatest need of it.

Of much greater value is it to secure a good night's rest, especially in women who are inclined to be nervous; and a sedative or hypnotic administered upon retiring will be of infinitely greater benefit to the patient than all the rectal cleansing in the world. Inquiries made in the operating theatres of those hospitals where the routine enema has been discarded have proved its unnecessary character in cases of ordinary radical cure of hernia.

### Operation.

Choice of operation must, of course, rest with the operator, who should always perform that with which he is most familiar, and which, to his own satisfaction, he has proved to produce the most reliable results. Where he has obtained equal experience and dexterity in two different but equally satisfactory operations, then he should rather choose to perform that with which the less post-operative bed occupation is required, as giving the greater benefit to the patients remaining upon the "waiting-list."

Probably the most satisfactory procedure is one that follows the principles of Kocher, in that it tends to displace or abolish the natural spot of peritoneal weakness, and to replace it by a portion of stiff, taut membrane over the inner abdominal ring, the weak spot in the muscular wall. A supplementary reinforcement of the musculature at those places which have most felt the undue stretching produced by the hernial sac and its contents will then complete the operation.

The Bassini and Halstead operations are less satisfactory, in that they do not displace the weak spot in the peritoneum from the neighbourhood of the ring, but deal only with the muscular walls after removal of the sac; whereas the fundamental factor in the production of hernia is far more likely to rest with the condition of the peritoneum at the upper end of the canal than to depend upon any defect in the abdominal musculature. A hernia cannot exist without a peritoneal fault, whilst weak muscles do not necessarily induce or even accompany the lesion.

Having decided upon the operation to be practised in the average case, it remains to ensure rapidity of execution by means of repeated assistance of other operators, and by operations upon the cadaver, until it becomes possible to complete the usual straightforward operation, without any hurry or undue haste, within fifteen minutes. Care in immediate clamping of all bleeding points is always a saving of time, and only exceeded as such by avoiding the vessels altogether or by clamping them prior to section. A dry field of operation must obtain equally throughout the operation, and upon closing the incision; or a scrotal hæmatoma will usually occur and cause considerable delay in the healing of the wound.

### Question of Dressings.

As far as dressings are concerned, probably the less applied the better; and a film of wool soaked in collodion for an adult, or a paste or a powder of iodoform and boracic for an infant—provided the latter be covered with a cradle and prevented from touching the wound—make the best applications.

In the case of a muscular adult who appears likely to struggle considerably upon coming round, it may be as well to fix a pad and bandage over the wound for the first twenty-four hours, carefully removing them at the end of that time, or as soon as all vomiting has ceased, so that no urinous dressings may surround and foul the wound, which is, at the same time, hidden from inspection. A scrotal pillow is of advantage for the first forty-eight hours as preventing the sagging of the scrotum and the drag upon the cord, which tend to set up or to increase capillary hæmorrhage into the cord and scrotum and about the wound, and so to delay healing. Much struggling is avoided, however, by the judicious administration of scopolamine, morphine, and atropine before the operation, and by a further dose of morphine and atropine after operation, where much pain and restlessness are present. It is of great importance to secure an adequate post-operative sleep, as in that way alone can shock be minimised and the powers of the patient's resistance be increased so as to secure sound healing of the wound by first intention; this is especially the case with nervous women, when there can rarely be occasion for withholding a narcotic upon the first night after operation.

### After-treatment.

Fluids alone should be given for the first twenty-four hours, followed by a diet of "slops" and of light solids for a further day or two. A purgative should be given upon the second night or third morning, all being well, though flatulence or griping may exceptionally demand a turpentine and asafœtida enema previous to this. Such enemata should not, however, be given as a routine measure, because the pain and shock attendant upon their administration are liable rather to hinder than to aid the patient's progress. An ounce of glycerine by the rectum is far more easily administered and produces infinitely less discomfort to the patient, besides providing excellent results without any appreciable shock.

(*To be continued.*)

